# Common laboratory parameters as predictors of prognosis in primary lung cancer

**DOI:** 10.3389/fonc.2025.1708848

**Published:** 2026-01-12

**Authors:** Mingchun Cai, Hao Chen, Zhengbo Yan, Xuehua He

**Affiliations:** 1 Department of Medical Record Management, Tongliang District People’s Hospital, Chongqing, China; 2 Department of General Practice, Tongliang District People’s Hospital, Chongqing, China; 3 Department of Clinical Laboratory, Tongliang District People’s Hospital, Chongqing, China

**Keywords:** nomogram, overall survival, prediction model, primary lung cancer, prognostic

## Abstract

**Background:**

The prognosis of patients with primary lung cancer remains poor. Therefore, this study aimed to develop and validate a predictive model to evaluate the overall survival (OS) of these patients.

**Methods:**

A retrospective analysis was conducted on the data of 1,308 patients with primary lung cancer who received treatment and follow-up at our hospital from 2016 to 2022. The entire cohort was randomly divided into a derivation cohort (70%, n=915) and a validation cohort (30%, n=393) in a 7:3 ratio. A prognostic nomogram was constructed using Cox-least absolute shrinkage and selection operator regression analysis to predict the OS probabilities at 1-, 3-, and 5-years. Kaplan–Meier curve and log-rank tests were used to analyze and compare OS among different patient subgroups. The model was comprehensively evaluated through the area under the receiver operating characteristic curve (AUC), calibration curves, and decision curve analysis (DCA).

**Results:**

Age, gender, red blood cell count, serum potassium, albumin-globulin ratio, and prothrombin time activity were the prognostic indicators for predicting OS in patients with primary lung cancer. In the derivation cohort, the AUCs at 1-, 3-, and 5-years were 0.739 (95% confidence interval [CI]: 0.702–0.776), 0.727 (95% CI: 0.690–0.764), and 0.675 (95% CI: 0.629–0.721). In the validation cohort, the AUCs at 1-, 3-, and 5-years were 0.770 (95% CI: 0.712–0.827), 0.784 (95% CI: 0.732–0.837), and 0.717 (95% CI: 0.646–0.789), respectively. The calibration curve and DCA results confirmed the model’s good predictive power.

**Conclusion:**

In this study, we developed and validated an OS prediction model for patients with primary lung cancer. Providing personalized predictions with multiple outcomes increases the information available to patients and clinicians.

## 1 Introduction

Lung cancer is one of the most prevalent malignant tumors globally. The latest research by the American Cancer Society projected that, by the end of 2022, there will be 1,918,030 new cancer cases and 609,360 cancer-related deaths in the United States (US), with approximately 350 deaths occurring daily from lung cancer (1). Lung cancer holds the highest mortality rate among malignant tumors worldwide and ranks first in both incidence and mortality rates in China (2). In 2020, there were 539,100 new cases, and 471,500 people died from lung cancer (3). Additionally, lung cancer is one of the primary causes of disability-adjusted life years (4, 5). The low 5-year survival rate of cancer compels clinicians to analyze and assess patients’ prognoses to optimize treatment strategies (6). Personalized prognostic diagnostics can guide tailored treatments, significantly advancing the development of precision medicine (7). Consequently, an accurate and practical prognostic model is essential.

Most current risk assessment models focus primarily on integrating disease-related dimensions, such as Tumor-Node-Metastasis (TNM) staging, imaging features, and specific pathological or genetic markers (8–10). Furthermore, several models have been developed based on routine laboratory indexes, such as inflammatory markers or albumin levels. However, models that systematically screen and integrate a broad panel of pre-treatment, routinely available laboratory parametersent,.DAT relying on specialized pathology, advanced imaging, or genomic datamic build a parsimonious and immediately deployable prognostic tool are less common. The novelty of this study lies in its exclusive focus on this universally accessible data domain. Its primary advantage over existing high-performance models is not necessarily superior predictive accuracy, but superior practicality and accessibility. It is designed as a “first-line” risk stratification tool that can be generated from a standard admission blood panel and basic demographics, providing rapid prognostic insight in settings where or at a time when detailed staging, advanced imaging (e.g., PET-CT), or genetic profiling results are not yet available. This addresses a distinct clinical gap where simplicity, speed, and broad applicability are paramount. Indeed, biomarkers and laboratory indicators play an indispensable role in facilitating rapid diagnosis and accurate prediction of short-term prognosis (11). Richlitzki et al. indicated that C-reactive protein (CRP) is a significant predictor of overall survival (OS) in patients with stage III non-small cell lung cancer (12). In a multicenter, observational, retrospective study (13), researchers developed a prognostic model designed to discern early differences between lung cancer and benign nodules, leveraging routine clinical and laboratory data. The XGBoost model demonstrated exceptional performance in differentiating advanced from early-stage lung cancer, achieving an area under the curve (AUC) as high as 0.913. Other laboratory parameters, including urea nitrogen, serum albumin, serum total protein, and neutrophil count, are also pivotal in the diagnostic and prognostic assessment of lung cancer (14–16). In addition to laboratory indicators, recent advancements in artificial intelligence (AI) have shown promise in improving diagnostic and prognostic accuracy in lung cancer (17–19). For instance, AI models have been applied to histopathological images for survival prediction and to CT imaging data for prognostication in non-small cell lung cancer. Furthermore, privacy-preserving distributed modeling approaches, such as federated learning, are being explored to develop robust prognostic models without compromising patient data privacy (20–22). These AI-driven methodologies, when integrated with conventional laboratory indicators, hold significant potential to enhance the accuracy of lung cancer diagnosis and prognosis.

While non-small cell lung cancer (NSCLC) and small cell lung cancer (SCLC) are distinct pathological subtypes with different prognoses and treatment paradigms, this study focused on primary lung cancer as a combined entity. This approach was taken for two primary reasons. First, our objective was to develop a pragmatic, initial risk-stratification tool based on universally available routine data prior to the completion of comprehensive pathological subtyping, which can sometimes be delayed in clinical practice. Second, we aimed to identify common, systemic physiological derangements (reflected in laboratory parameters) that influence prognosis across the spectrum of lung cancer, thereby providing a broadly applicable clinical instrument.

Therefore, this study compiled a large set of routine laboratory data from patients with lung cancer, aiming to investigate the risk factors affecting their prognosis and to develop a prognostic prediction model to assist in clinical decision-making. This approach will enable physicians to more accurately assess patients’ conditions and provide a basis for implementing personalized treatment plans.

## 2 Methods

### 2.1 Study design and patients

This study adhered to the Transparent Reporting of a Multivariable Prediction Model for Individual Prognosis or Diagnosis (TRIPOD) guidelines. All patients with lung cancer admitted to the People’s Hospital of Tongliang District between January 1, 2016, and December 31 2022, were included in this study. The entire cohort of 1,308 patients was then randomly divided into a derivation cohort (70%, n=915) and an internal validation cohort (30%, n=393) using a computer-generated random sequence. This split was performed to ensure independent assessment of the model’s performance. This study was approved by the Ethics Committee of the People’s Hospital of Tongliang District (Ethical Approval No. TLLLKY2023041) and was conducted following the Declaration of Helsinki. Informed consent for participation was not required due to the retrospective design of the study, which complied with national legislation and institutional requirements.

### 2.2 Inclusion and exclusion criteria

**Figure 1:**
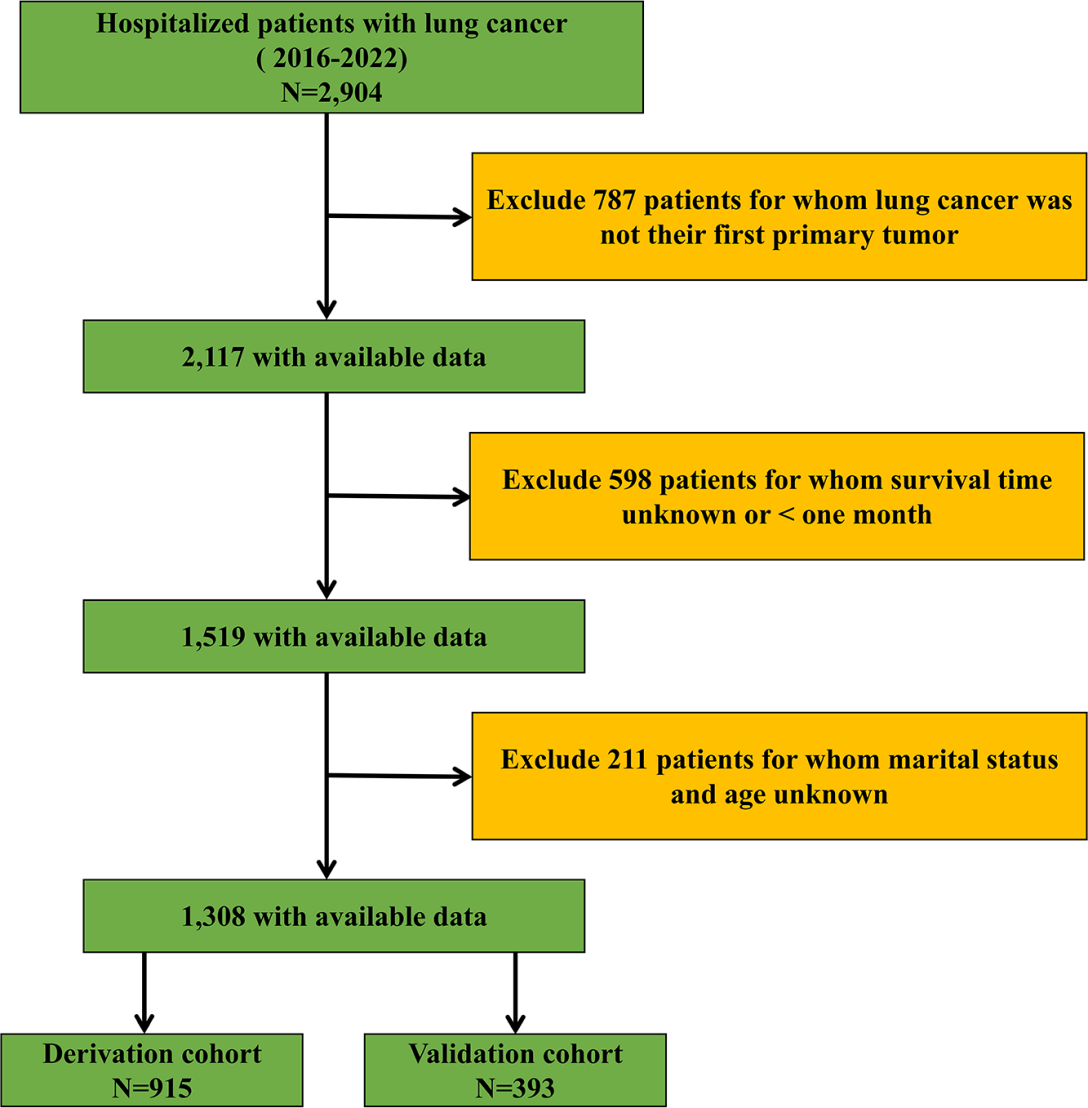
Flow diagram of the study design.

The inclusion criteria were as follows: (i) Age ≥ 18 years and (ii) a diagnosis of lung cancer confirmed through histopathological examination. The exclusion criteria were as follows: (i) Lung cancer not being the first primary cancer; (ii) survival time unknown or < one month; (iii) unknown marital status and age. The inclusion and exclusion criteria are outlined in **Figure 1**.

### 2.3 Data collection

A total of 26 candidate variables were selected. All laboratory indicators were collected as baseline measurements, specifically from the first complete blood tests performed at the time of or immediately following the confirmed diagnosis of primary lung cancer, and prior to the initiation of any definitive anti-cancer treatment (such as surgery, chemotherapy, radiotherapy, or targeted therapy). This approach ensured that the variables reflected the patient’s pre-treatment physiological state. Specifically, we explored age, gender, marital status, white blood cell (WBC) count, red blood cell (RBC) count, neutrophil-lymphocyte ratio (NLR), lymphocyte-monocyte ratio (LMR), platelet-lymphocyte ratio (PLR), serum creatinine, serum chloride, serum potassium, serum sodium, serum calcium, serum phosphorus, serum magnesium, basophil percentage, eosinophil percentage, total bilirubin (TBIL), direct bilirubin (DBIL), albumin-globulin ratio (AGR), aspartate aminotransferase/alanine aminotransferase (AST/ALT), alkaline phosphatase (ALP), urea, uric acid (UA), prothrombin time activity (PTA), and hypersensitive C-reactive protein (hs-CRP).

### 2.4 Statistical analyses

Statistical analyses were performed using the Statistical Package for the Social Sciences (version 22.0) and R (version 4.3.3) software. Categorical data are expressed as frequencies and percentages, and the chi-square test was used for group comparisons. Continuous data with a normal distribution are expressed as mean ± standard deviation, and intergroup differences were analyzed using the t-test. For continuous data that did not follow a normal distribution, the median and interquartile range (M [Q1-Q3]) were reported, and comparisons were made using the Mann–Whitney U test. To handle missing data and maximize statistical power, we employed multivariate multiple imputations with chained equations (MICE). We performed 10 imputations, and convergence was assessed using the Gelman-Rubin statistic, with values close to 1 indicating satisfactory convergence.

The Cox-least absolute shrinkage and selection operator (LASSO) regression analysis was used to identify independent prognostic predictors. The optimal λ value was determined using the one-standard-error rule, which selects the largest λ value within one standard error of the minimum cross-validation error. Based on the results of multivariate Cox regression analysis, independent risk predictors were utilized to create a prognostic nomogram to predict the probability of OS at 1-, 3-, and 5-years. The score assignment for each indicator in the nomogram was calculated by assigning each predictor variable a score based on its hazard ratio (HR) from the Cox regression model, summing the individual scores of all predictor variables to obtain a total score for each patient, and then mapping this total score to the corresponding survival probabilities at 1-, 3-, and 5-years using the nomogram. The Kaplan–Meier (KM) curve and log-rank tests were used to analyze and compare the OS of patients in different subgroups. The model was comprehensively evaluated through the area under the receiver operating characteristic (ROC) curve (AUC), calibration curves, and decision curve analysis (DCA). To further validate our model, we compared it with two other machine learning-based survival models: Random Survival Forests (RSF) and XGBoost survival analysis. These models were implemented using the R packages ‘randomForestSRC’ and ‘xgboost’, respectively. The performance of these models was evaluated using the same metrics as our Cox-LASSO model. All statistical tests were two-tailed, with a significance level set at *P* < 0.05.

## 3 Results

### 3.1 Patient characteristics

A total of 1,308 patients with primary lung cancer selected through stringent screening procedures and inclusion/exclusion criteria were included in this study. The patients were divided into derivation and validation cohorts in a 7:3 ratio, comprising 915 patients in the derivation cohort and 393 in the validation cohort. Among these, 957 were males (73.17%) and 351 were females (26.83%). Most patients were married (85.24%). **Table 1** provides a detailed summary of the basic information of the patients with primary lung cancer included in this study. The results of the multiple imputation for missing data are detailed in **Supplementary Tables 1**, **Supplementary Table 2**. These results indicate that, in both the derivation and validation cohorts, there were no statistically significant differences in the imputed values for all missing indicators before and after imputation.

**Table 1:** Baseline characteristics of the patients.

Variables	Total (N = 1,308)	Derivation cohort (N = 915)	Validation cohort (N = 393)	*P* value
Age (years)	66.57 ± 9.92	66.25 ± 10.00	67.32 ± 9.70	0.074
Gender				0.133
Female	351 (26.83%)	234 (25.57%)	117 (29.77%)	
Male	957 (73.17%)	681 (74.43%)	276 (70.23%)	
Marital status				0.135
Married/Living with partner	1,115 (85.24%)	778 (85.03%)	337 (85.75%)	
Never married	50 (3.83%)	41 (4.48%)	9 (2.29%)	
Widowed/Divorced/Separated	143 (10.93%)	96 (10.49%)	47 (11.96%)	
WBC count (× 10^9^/L)	7.28 (5.60, 9.29)	7.33 (5.71, 9.25)	7.03 (5.36, 9.46)	0.215
RBC count (×10^12^/L)	4.04 ± 0.63	4.05 ± 0.62	4.01 ± 0.65	0.354
NLR	4.60 (3.08, 7.31)	4.65 (3.18, 7.42)	4.54 (2.75, 7.12)	0.090
LMR	2.53 (1.69, 3.78)	2.42 (1.64, 3.75)	2.74 (1.87, 3.85)	0.014
PLR	198.95 (141.13, 301.12)	206.67 (146.25, 312.66)	182.44 (132.41, 282.00)	0.005
Serum creatinine (μmol/L)	59.00 (40.55, 72.40)	58.70 (33.50, 71.80)	59.60 (43.80, 73.50)	0.155
Serum chloride (mmol/L)	102.81 ± 4.87	102.78 ± 4.84	102.86 ± 4.93	0.800
Serum potassium (mmol/L)	4.06 ± 0.50	4.05 ± 0.50	4.07 ± 0.49	0.650
Serum sodium (mmol/L)	139.99 ± 4.13	139.94 ± 4.00	140.09 ± 4.43	0.546
Serum calcium (mmol/L)	2.13 ± 0.26	2.13 ± 0.24	2.14 ± 0.29	0.326
Serum phosphorus (mmol/L)	1.06 ± 0.24	1.06 ± 0.24	1.05 ± 0.21	0.725
Serum magnesium (mmol/L)	0.81 ± 0.10	0.81 ± 0.11	0.81 ± 0.10	0.717
Basophil percentage (%)	0.40 (0.20, 0.60)	0.40 (0.20, 0.60)	0.40 (0.20, 0.50)	0.372
Eosinophil percentage (%)	2.00 (0.90, 3.80)	2.00 (0.90, 3.90)	2.00 (0.80, 3.50)	0.504
TBIL (umol/L)	10.40 (7.50, 13.83)	10.30 (7.40, 13.50)	10.60 (7.90, 14.70)	0.144
DBIL (umol/L)	3.40 (2.50, 4.70)	3.40 (2.50, 4.60)	3.50 (2.50, 4.80)	0.261
AGR	1.46 ± 0.33	1.46 ± 0.33	1.46 ± 0.34	0.701
AST/ALT	1.20 (0.90, 1.60)	1.20 (0.90, 1.50)	1.30 (1.00, 1.70)	0.006
ALP (U/L)	82.00 (66.80, 103.83)	82.10 (67.00, 103.95)	81.90 (65.70, 102.90)	0.944
Urea (mmol/l)	5.51 ± 2.34	5.47 ± 2.46	5.61 ± 2.04	0.323
UA (umol/L)	278.64 ± 99.03	279.44 ± 101.09	276.77 ± 94.02	0.655
PTA (%)	100.48 ± 20.48	100.67 ± 20.20	100.04 ± 21.10	0.611
hs-CRP (mg/L)	13.91 (4.34, 47.48)	16.00 (5.00, 47.83)	12.00 (3.50, 45.00)	0.069

WBC, white blood cell; RBC, red blood cell; NLR, neutrophil-lymphocyte ratio; LMR, lymphocyte-monocyte ratio; PLR, platelet-lymphocyte ratio; TBIL, total bilirubin; DBIL, direct bilirubin; AGR, albumin–globulin ratio; AST, aspartate aminotransferase; ALT, alanine aminotransferase; ALP, alkaline phosphatase; UA, uric acid; PTA, prothrombin time activity; hs-CRP, hypersensitive C-reactive protein.

### 3.2 Identification of the predictive factors for OS

**Figure 2:**
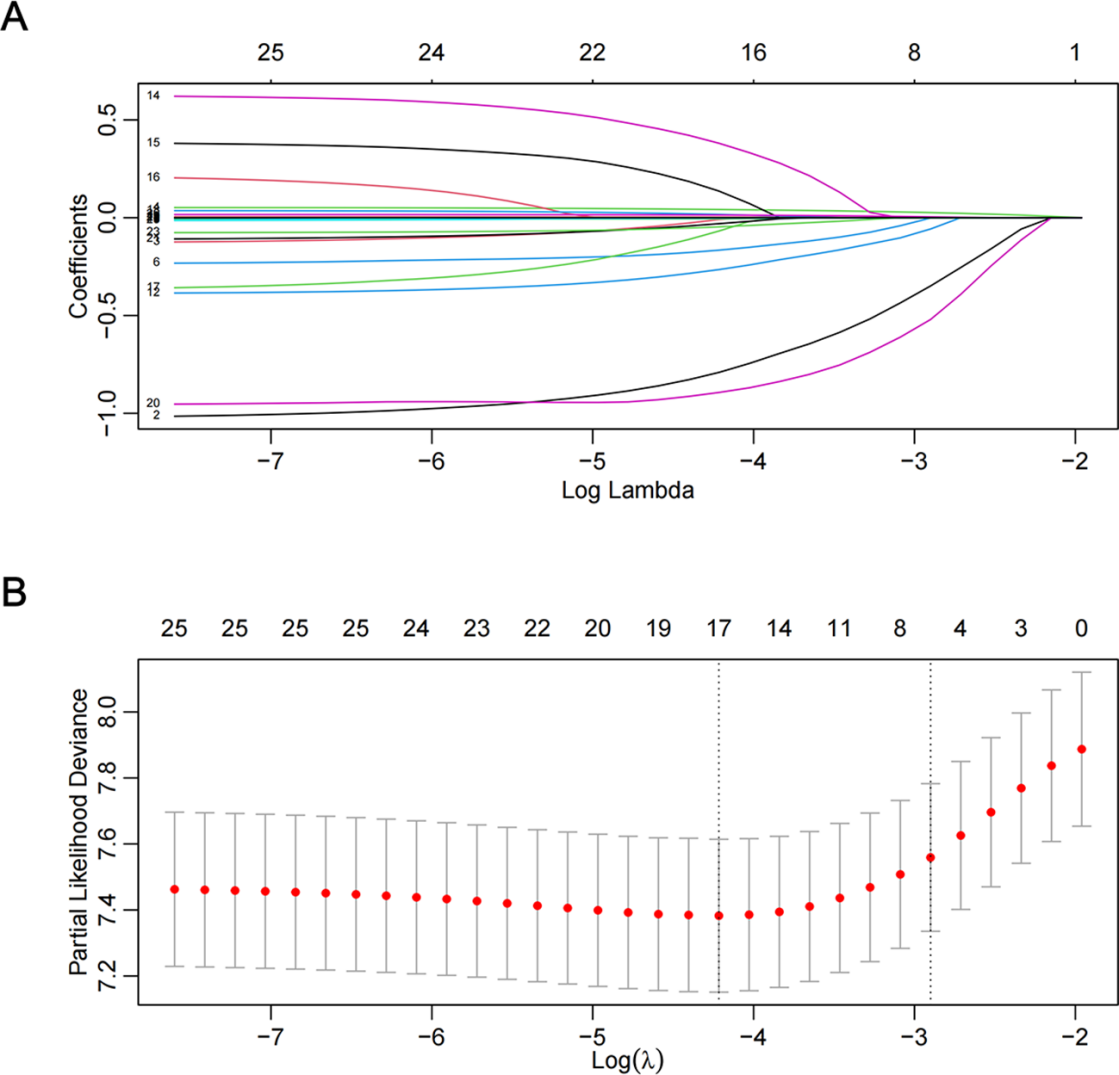
Feature selection by Cox-LASSO. **(A)** Cox-LASSO coefficient profiles (y-axis) of features; **(B)** 10-fold cross-validation for tuning parameter selection in the Cox-LASSO model.

**Figure 3:**
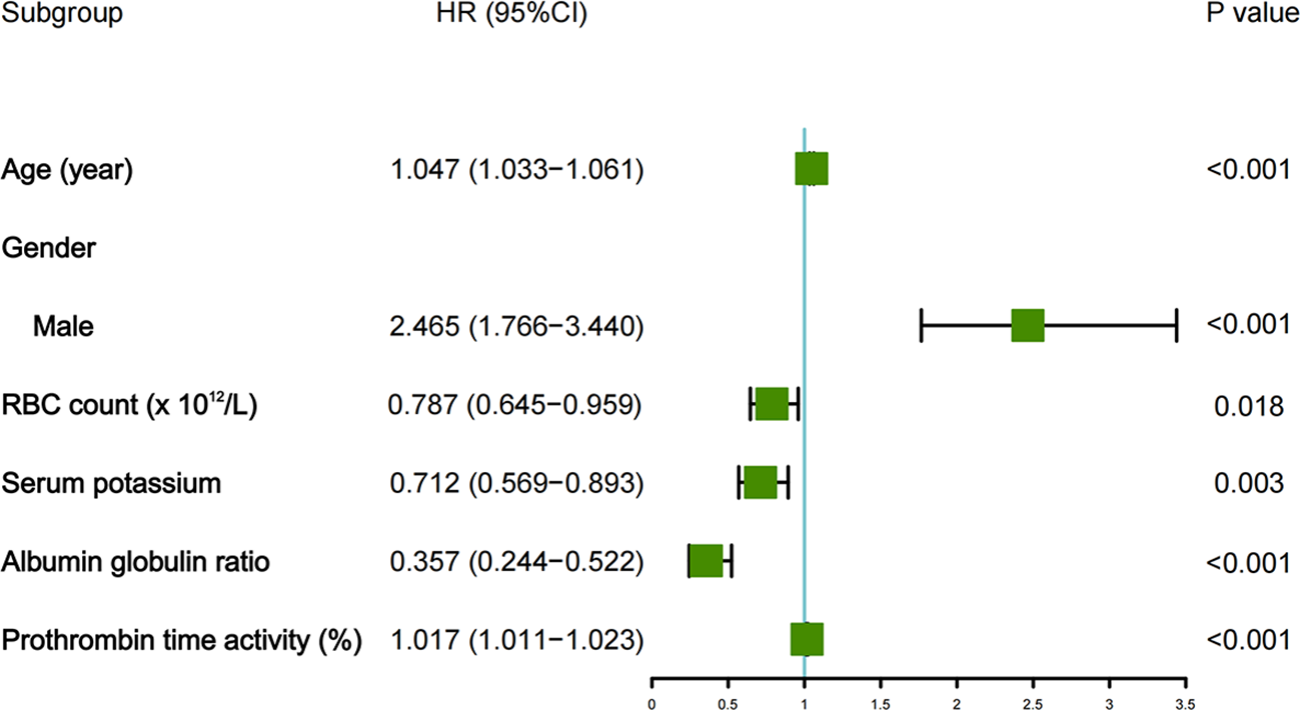
Forest plot displaying the results of the multivariable Cox analysis.

As displayed in **Figure 2**, the Cox-LASSO model selected lambda.1se, corresponding to a λ value of 0.05501995, and six predictors: age, gender, RBC count, serum potassium, AGR, and PTA. The multivariate Cox regression model indicated that age (HR = 1.047, 95% CI: 1.033atednte and *P* < 0.001), gender (male) (HR = 2.465, 95% CI: 1.766)tednte and *P* < 0.001), RBC count (HR = 0.787, 95% CI: 0.645,tednte and P = 0.018), serum potassium (HR = 0.712, 95% CI: 0.569siumnte and *P* = 0.003), AGR (HR = 0.357, 95% CI: 0.244,iumnte and *P* < 0.001), and PTA (HR = 1.017, 95% CI: 1.011,iumnte and *P* < 0.001) were the prognostic indicators for predicting OS in patients with primary lung cancer (**Figure 3**).

### 3.3 Development of prediction model for OS

**Figure 4:**
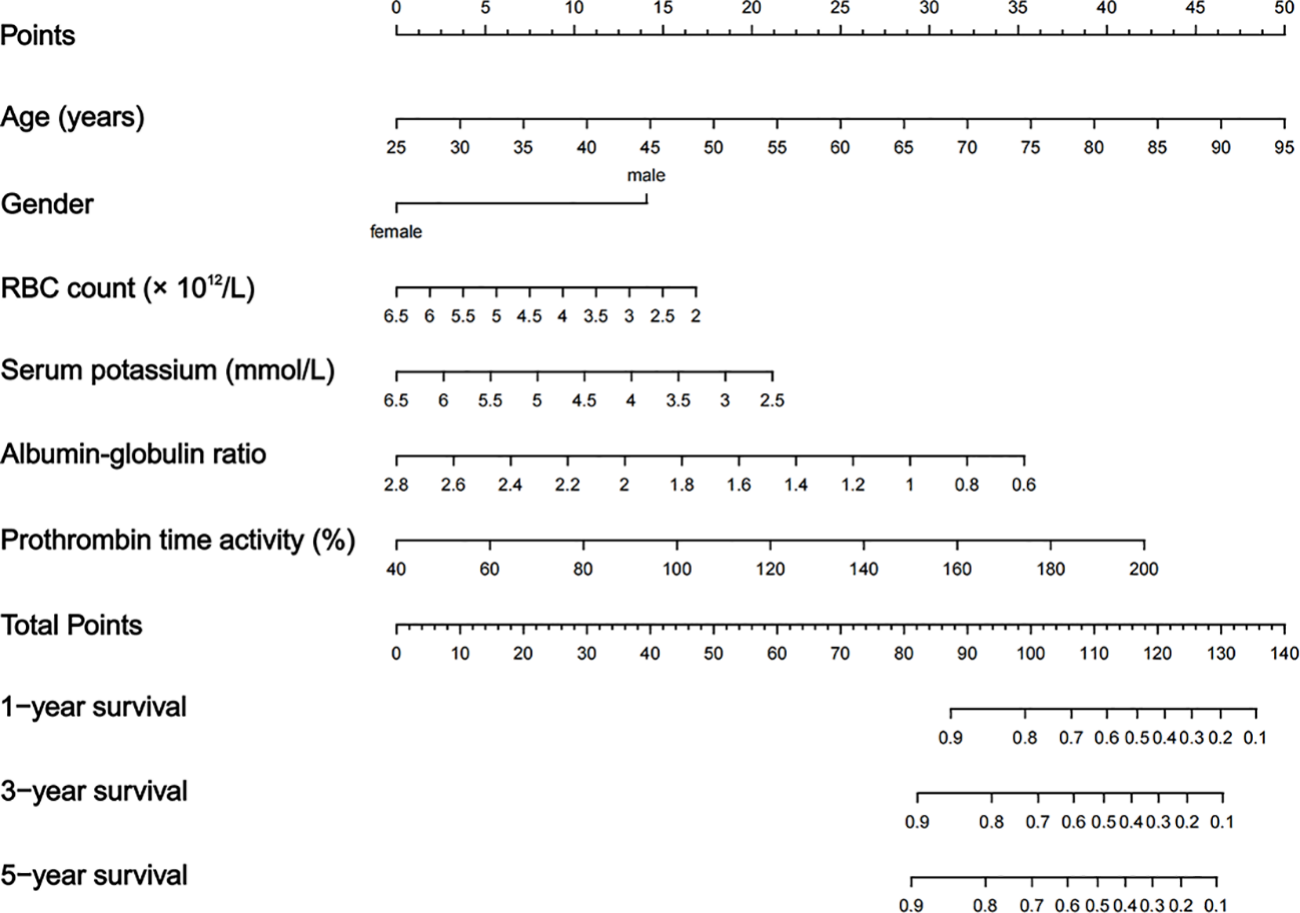
Nomogram predicting 1-, 3-, and 5-years OS.

We developed a nomogram for OS. Each variable was assigned a point based on HR. Then, by summing the total score of each variable and locating the score on the total points scale, we obtained a nomogram predicting 1-, 3-, and 5-year OS. The nomogram containing independent predictive factors for predicting 1-, 3-, and 5-year OS of patients with primary lung cancer is illustrated in **Figure 4**.

### 3.4 Performance of the prediction model for OS

**Figure 5:**
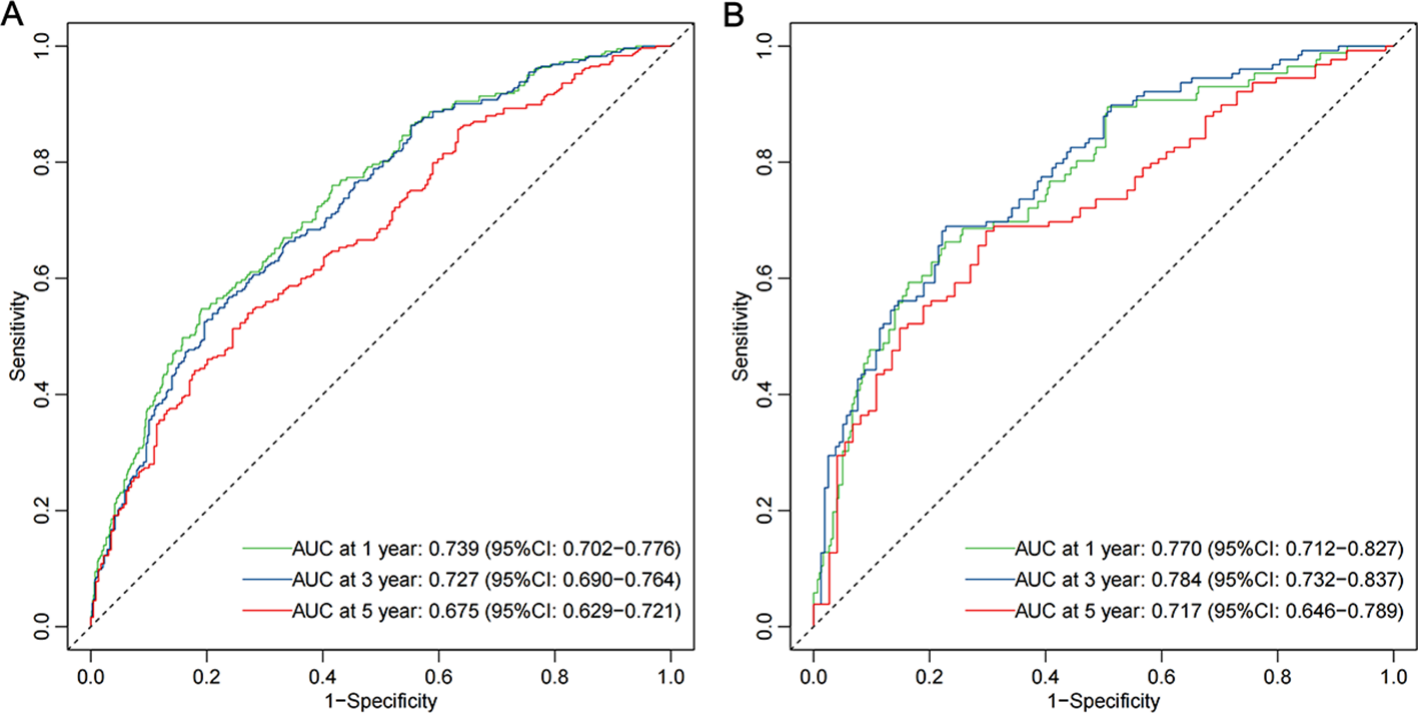
ROC curves of the model. **(A)** Derivation cohort; **(B)** Validation cohort.

**Figure 6:**
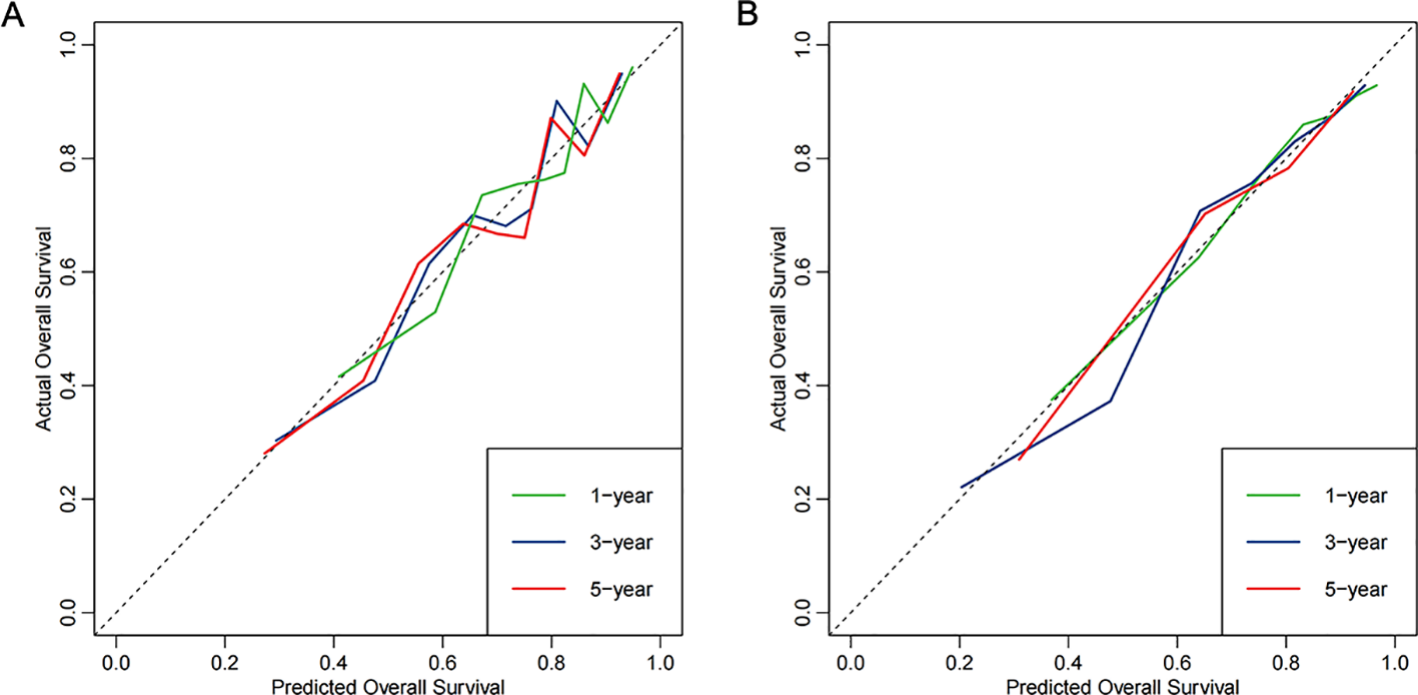
Calibration curves of the model. **(A)** Derivation cohort; **(B)** Validation cohort.

**Figure 7:**
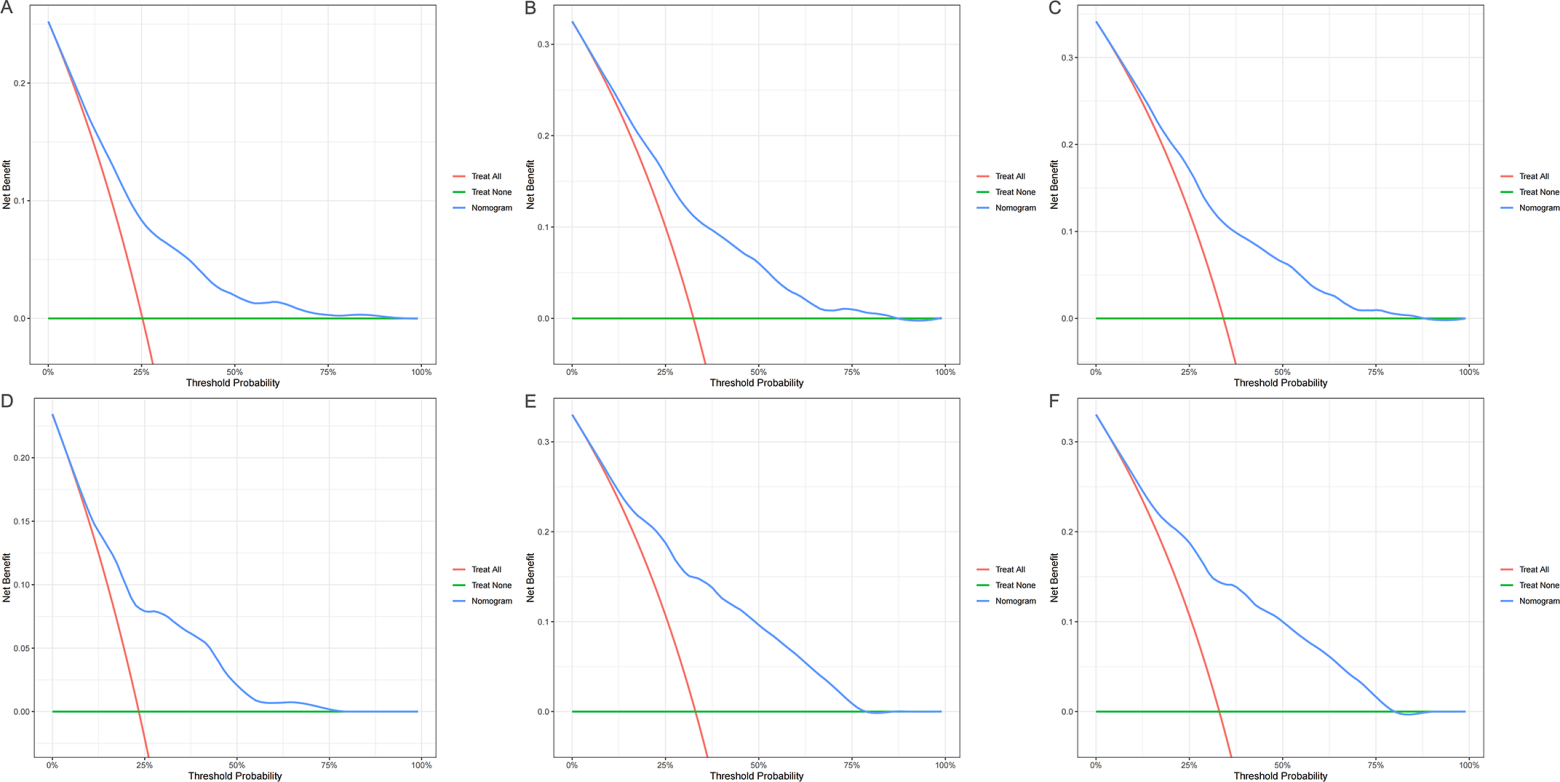
Decision curves of the model. **(A)** 1-year in the derivation cohort; **(B)** 3-year in the derivation cohort; **(C)** 5-year in the derivation cohort; **(D)** 1-year in the validation cohort; **(E)** 3-year in the validation cohort; **(F)** 5-year in the validation cohort.

The time-dependent ROC curves demonstrated that the AUCs at 1-, 3-, and 5-years were 0.739 (95% CI: 0.702–0.776), 0.727 (95% CI: 0.690–0.764), and 0.675 (95% CI: 0.629–0.721), respectively, in the derivation cohort. In the validation cohort, the AUCs at 1-, 3-, and 5-years were 0.770 (95% CI: 0.712–0.827), 0.784 (95% CI: 0.732–0.837), and 0.717 (95% CI: 0.646–0.789), respectively (**Figures 5A, B**). Additionally, calibration curves (**Figures 6A, B**) indicated that the nomogram has good predictive accuracy in both the derivation and validation cohorts. The DCA curves (**Figures 7A–F**) also revealed that the nomogram has good clinical utility in both cohorts. To further evaluate our model, we compared it with RSF and XGBoost survival analysis models. The AUCs for the RSF model at 1-, 3-, and 5-years were 0.745, 0.725, and 0.673, respectively (**Supplementary Figure 1**). For the XGBoost model, the AUCs at 1-, 3-, and 5-years were 0.716, 0.660, and 0.591, respectively (**Supplementary Figure 1**). Collectively, these results indicate that our Cox-LASSO model performs comparably to these machine learning models, demonstrating a slight advantage at certain time points. To enhance the interpretability of our model, we have included a visualization of feature importance based on the coefficients from our Cox-LASSO model (**Supplementary Figure 2**).

### 3.5 Sensitivity analysis

To further validate the stability of our predictive model, we conducted a sensitivity analysis based on marital status. The model was evaluated in three subgroups: Married/Living with partner, Never married, and Widowed/Divorced/Separated. The results showed that the model maintained good predictive performance across all subgroups, with AUCs ranging from 0.650 to 0.750 at 1-, 3-, and 5-years (**Supplementary Figure 3**). This analysis demonstrates the robustness of our model in different demographic subgroups.

We observed differences in LMR, PLR, and AST/ALT between the derivation and validation cohorts. To ensure that these differences do not affect the stability of our predictive model, we conducted sensitivity analyses. We re-verified the model after excluding patients with abnormal indicators (LMR, PLR, and AST/ALT outside the normal range). The results showed that the model maintained good predictive performance, with AUCs at 1-, 3-, and 5-years remaining stable (**Supplementary Figure 4**). This analysis confirms that the model’s stability is not significantly affected by the observed differences in these indicators.

### 3.6 KM survival analysis

**Figure 8:**
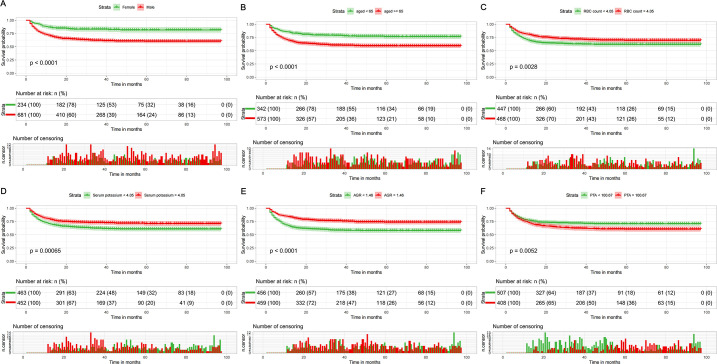
KM curves for the predictors. **(A)** Gender; **(B)** Age; **(C)** RBC count; **(D)** Serum potassium; **(E)** AGR; **(F)** PTA.

We conducted survival analysis on the six prognostic factors separately. The results of the KM continuity study revealed that the OS of male patients was significantly lower than that of female patients (*P* < 0.001), while patients aged < 65 years exhibited a considerably higher OS (*P* < 0.001) (**Figures 8A, B**). Additionally, we observed reduced OS in patients with lower RBC counts (*P* = 0.003), lower serum potassium levels (*P* < 0.001), lower AGR (*P* < 0.001), and higher PTA (*P* = 0.005) (**Figures 8C–F**).

## 4 Discussion

In this study, we evaluated several prognostic variables associated with OS in patients with primary lung cancer. Our findings indicated that a straightforward prediction model based on six prognostic factors—age, gender, RBC count, serum potassium, AGR, and PTA—can effectively predict the 1-, 3-, and 5-year OS of these patients, with AUCs of 0.739 (95% CI: 0.702–0.776), 0.727 (95% CI: 0.690–0.764), and 0.675 (95% CI: 0.629–0.721), respectively.

Advanced age is a significant risk factor affecting the long-term survival of patients with primary lung cancer. The poor prognosis in elderly patients is associated with digestive dysfunction, reduced physiological reserve, poor tolerance to surgery and chemotherapy, and the presence of multiple comorbidities (23–25). Chen et al. (26) demonstrated significant differences in clinical characteristics and prognosis among patients with lung cancer across various age groups. Compared to patients aged 40 years or younger, those over 80 years have a higher risk of mortality. Huh et al. (27) also indicated that one of the strongest risk factors for lung cancer mortality was age ≥ 65 years.

Gender significantly impacts the survival status of patients with primary lung cancer. In this study, the mortality rate among male patients was much higher than that among female patients (1-year mortality rate: male to female ratio of 1.014:1; 3-year mortality rate: male to female ratio of 1.603:1; 5-year mortality rate: male to female ratio of 1.923:1), which was also confirmed in a cohort from the US (28). A nationwide cohort study of non-small cell lung cancer in Switzerland also demonstrated that male survival rates were significantly worse than those of females at various clinical stages (29). Another study from Korea indicated that female gender is a better prognostic factor for patients with small cell lung cancer, even after comprehensive adjustment for all prognostic variables (adjusted HR: 0.51, 95% CI: 0.34–0.77) (30). The significant gender disparity in survival observed in our cohort (male: 73.17%) aligns with these global trends but must be interpreted considering potential local confounders. The pronounced male predominance in our study population may reflect, in part, regional differences in smoking prevalence and occupational exposures. Furthermore, while our multivariable model adjusted for key laboratory parameters, residual confounding from unmeasured factors—such as detailed smoking history, specific treatment modalities received (e.g., types of surgery or chemotherapy regimens), socio-economic status, and comorbidities—could contribute to the observed association between male gender and poorer prognosis. Future studies with more granular data are needed to disentangle the independent effect of gender from these closely linked lifestyle and clinical factors. The higher mortality rate in males may be related to adverse habits such as smoking and drinking (31, 32). Conversely, females are less likely to smoke and drink than males, resulting in lower exposure to risk factors in terms of both time and intensity.

Our study identified four common laboratory indicators (RBC count, serum potassium, AGR, and PTA) that can serve as prognostic variables for OS in patients with primary lung cancer. RBCs are responsible for transporting oxygen to various parts of the body. A low RBC count can limit oxygen supply, potentially influencing tumor growth rate and invasiveness, thereby affecting treatment efficacy and patient survival (33). Furthermore, a low RBC count may be associated with chronic inflammatory conditions, where the inflammatory microenvironment can promote tumor development and metastasis (34). Regarding serum potassium, while its level is tightly regulated, variations within the normal range may reflect underlying metabolic or nutritional status. Our finding that lower serum potassium is associated with worse prognosis warrants further investigation into its potential role in the context of cancer cachexia or treatment-related metabolic disturbances (35, 36). Numerous studies have examined the role of the AGR in lung cancer prognosis. Yao et al. (37) demonstrated a significant correlation between pre-treatment AGR and patients with advanced non-small cell lung cancer. Duran et al. (38) identified low AGR as a strong predictor of long-term mortality risk in patients with lung adenocarcinoma. Zhou et al. indicated that an AGR below 1.29 was an independent predictor of OS deterioration in patients with small-cell lung cancer (39). Patients with late-stage lung cancer often exhibit dysfunctions in coagulation and fibrinolysis systems, with chemotherapy potentially exacerbating these abnormalities (40). Changes in fibrinolysis parameters are particularly sensitive indicators of such exacerbations. PTA is a crucial marker for assessing coagulation function, and an elevated PTA may indicate the activation status of the coagulation system.

In addition to laboratory indicators, multimodal approaches that integrate laboratory and imaging data have shown promise in improving diagnostic and prognostic accuracy in thoracic oncology. For instance, Rani et al. proposed a multi-modal bone suppression, lung segmentation, and classification approach for accurate COVID-19 detection using chest radiographs (41). This approach demonstrates the potential of integrating advanced image preprocessing pipelines with laboratory data to enhance diagnostic accuracy. Similarly, Rani et al. introduced a spatial feature and resolution maximization GAN for bone suppression in chest radiographs, which further highlights the importance of multimodal data integration in thoracic oncology (42). These studies suggest that combining laboratory indicators with advanced imaging techniques can provide a more comprehensive assessment of patient prognosis.

The primary innovation of this study lies in its methodological focus and pragmatic design. Unlike models that depend on specialized or delayed diagnostic information, we systematically derived and validated a parsimonious prognostic tool using exclusively pre-treatment, routine laboratory data and basic demographics. This approach identifies a core set of systemic physiological markers (RBC, potassium, AGR, PTA) that provide independent prognostic value across a broad lung cancer population. Clinically, this model offers several practical implications. First, it serves as an immediate, accessible risk stratification instrument that can be calculated at the time of initial diagnosis, providing early prognostic insight to clinicians and patients while awaiting more definitive staging and molecular profiling. Second, by highlighting parameters like AGR and PTA, it draws attention to the prognostic relevance of nutritional-inflammation status and coagulation function, which may be modifiable therapeutic targets. Finally, the nomogram format facilitates personalized communication of survival probabilities at 1, 3, and 5 years, aiding in shared decision-making and the tailoring of follow-up intensity. While not a replacement for comprehensive staging, this tool adds a valuable, rapid-assessment layer to the clinical management pathway.

Several limitations should be noted: (i) Although the present study is based on real-world data, the inherent nature of a single-center retrospective analysis introduces potential bias, necessitating further validation through multicenter prospective studies; (ii) we did not include several critical prognostic variables, most notably the tumor stage, detailed histology (NSCLC vs. SCLC subtype), specific genetic mutation status (e.g., EGFR, ALK), and the treatment modality received by the patient (e.g., surgery, type of chemotherapy/immunotherapy, radiotherapy). We recognize that this omission is a major limitation, as these factors are fundamental determinants of prognosis in lung cancer. Consequently, the reliability and clinical applicability of our current model are indeed constrained; it is not intended to replace comprehensive staging and molecular-based prognostic systems. Instead, its utility lies as a preliminary, complementary risk-assessment tool that leverages universally available data. Future studies integrating these crucial clinical-pathological variables with the baseline laboratory parameters are essential to develop a more robust and comprehensive predictive model; (iii) our study did not conduct repeated measurements at different time points to observe the dynamic changes of these laboratory parameters. Given the potential value of dynamic monitoring, future research should focus on collecting longitudinal data to explore the association between changes in laboratory parameters (e.g., the decrease range of serum potassium level) and OS. This approach could provide a more nuanced understanding of disease progression and improve the accuracy of prognostic models. To address some of these limitations, we conducted a sensitivity analysis based on marital status, which showed that the model maintained good predictive performance across different subgroups. To address the baseline differences observed in LMR, PLR, and AST/ALT between the two cohorts, we conducted a supplementary analysis and sensitivity analyses. The differences may be attributed to variations in patient inclusion time and treatment plans. Our sensitivity analyses confirmed that the model’s stability is not significantly affected by these differences, ensuring the robustness of our predictive model.

## 5 Conclusion

This population-based study identified independent predictive factors associated with OS in patients with primary lung cancer. Additionally, we developed and validated a novel, robust, and reliable predictive model that provide more accurate individualized survival estimates and can be utilized for clinical counseling.

## Data Availability

The raw data supporting the conclusions of this article will be made available by the authors, without undue reservation.
