# Supplementary material for: Common laboratory parameters as predictors of prognosis in primary lung cancer
Source: Front Oncol. 2026 Jan 12;15:1708848. doi: 10.3389/fonc.2025.1708848 (PMC12832266; doi:10.3389/fonc.2025.1708848)

**Table S1.** Comparison of missing variables in the derivation cohort before and after multiple imputation

| **Variables** | **Before interpolation** | **After interpolation** | ***P* value** |
| --- | --- | --- | --- |
| WBC count (× 10^9/L) | 7.08 (5.35, 9.59) | 7.33 (5.71, 9.25) | 0.932 |
| RBC count (×10^12/L) | 4.02±0.65 | 4.05±0.62 | 0.872 |
| NLR | 4.53 (2.74, 7.05) | 4.65 (3.18, 7.42) | 0.946 |
| LMR | 2.75 (1.86, 3.93) | 2.42 (1.64, 3.75) | 0.860 |
| PLR | 181.55 (131.46, 281.13) | 206.67 (146.25, 312.66) | 0.921 |
| Serum creatinine (μmol/L) | 59.65 (43.88, 74.05) | 58.70 (33.50, 71.80) | 0.870 |
| Serum chloride (mmol/L) | 102.81±4.95 | 102.78±4.84 | 0.858 |
| Serum potassium (mmol/L) | 4.07±0.48 | 4.05±0.50 | 0.888 |
| Serum sodium (mmol/L) | 140.12±4.37 | 139.94±4.00 | 0.766 |
| Serum calcium (mmol/L) | 2.14±0.29 | 2.13±0.24 | 0.559 |
| Serum phosphorus (mmol/L) | 1.05±0.22 | 1.06±0.24 | 0.982 |
| Serum magnesium (mmol/L) | 0.81±0.09 | 0.81±0.11 | 0.911 |
| Basophil percentage (%) | 0.40 (0.20, 0.50) | 0.40 (0.20, 0.60) | 0.954 |
| Eosinophil percentage (%) | 2.00 (0.90, 3.50) | 2.00 (0.90, 3.90) | 0.973 |
| TBIL (umol/L) | 10.60 (7.90, 14.70) | 10.30 (7.40, 13.50) | 0.956 |
| AGR | 1.46±0.33 | 1.46±0.33 | 0.983 |
| AST/ALT | 1.30 (1.00, 1.70) | 1.20 (0.90, 1.50) | 0.813 |
| ALP (U/L) | 81.65 (64.50, 101.73) | 82.10 (67.00, 103.95) | 0.923 |
| Urea (mmol/l) | 5.63±2.03 | 5.47±2.46 | 0.948 |
| UA (umol/L) | 277.63±94.50 | 279.44±101.09 | 0.939 |
| DBIL (umol/L) | 3.50 (2.50, 4.80) | 3.40 (2.50, 4.60) | 0.989 |
| PTA (%) | 100.32±20.96 | 100.67±20.20 | 0.959 |
| hs-CRP (mg/L) | 12.78 (3.80, 45.75) | 16.00 (5.00, 47.83) | 0.800 |

***WBC****: white blood cell;* ***RBC****: red blood cell;* ***NLR****: neutrophil-lymphocyte ratio;* ***LMR****: lymphocyte-monocyte ratio;* ***PLR****: platelet-lymphocyte ratio;* ***TBIL****: total bilirubin;* ***DBIL****: direct bilirubin;* ***AGR****: albumin–globulin ratio;* ***AST****: aspartate aminotransferase;* ***ALT****: alanine aminotransferase;* ***ALP****: alkaline phosphatase;* ***UA****: uric acid;* ***PTA****: prothrombin time activity;* ***hs-CRP****: hypersensitive C-reactive protein.*

**Table S2.** Comparison of missing variables in the validation cohort before and after multiple imputation

| **Variables** | **Before interpolation** | **After interpolation** | ***P* value** |
| --- | --- | --- | --- |
| WBC count (× 10^9/L) | 7.36 (5.71, 9.24) | 7.03 (5.36, 9.46) | 0.895 |
| RBC count (×10^12/L) | 4.04±0.61 | 4.01±0.65 | 0.896 |
| NLR | 4.64 (3.17, 7.41) | 4.54 (2.75, 7.12) | 0.893 |
| LMR | 2.42 (1.66, 3.77) | 2.74 (1.87, 3.85) | 0.952 |
| PLR | 205.06 (146.25, 308.11) | 182.44 (132.41, 282.00) | 0.820 |
| Serum creatinine (μmol/L) | 58.30 (33.25, 71.58) | 59.60 (43.80, 73.50) | 0.984 |
| Serum chloride (mmol/L) | 102.81±4.87 | 102.86±4.93 | 0.894 |
| Serum potassium (mmol/L) | 4.05±0.50 | 4.07±0.49 | 0.977 |
| Serum sodium (mmol/L) | 139.98±4.00 | 140.09±4.43 | 0.967 |
| Serum calcium (mmol/L) | 2.12±0.25 | 2.14±0.29 | 0.918 |
| Serum phosphorus (mmol/L) | 1.06±0.25 | 1.05±0.21 | 0.838 |
| Serum magnesium (mmol/L) | 0.81±0.11 | 0.81±0.10 | 0.838 |
| Basophil percentage (%) | 0.40 (0.20, 0.60) | 0.40 (0.20, 0.50) | 0.991 |
| Eosinophil percentage (%) | 2.00 (0.90, 3.80) | 2.00 (0.80, 3.50) | 0.918 |
| TBIL (umol/L) | 10.30 (7.40, 13.50) | 10.60 (7.90, 14.70) | 0.922 |
| AGR | 1.46±0.33 | 1.46±0.34 | 0.983 |
| AST/ALT | 1.20 (0.90, 1.60) | 1.30 (1.00, 1.70) | 0.816 |
| ALP (U/L) | 81.90 (66.88, 103.93) | 81.90 (65.70, 102.90) | 0.804 |
| Urea (mmol/l) | 5.46±2.32 | 5.61±2.04 | 0.824 |
| UA (umol/L) | 278.74±99.32 | 276.77±94.02 | 0.888 |
| DBIL (umol/L) | 3.40 (2.50, 4.60) | 3.50 (2.50, 4.80) | 0.980 |
| PTA (%) | 100.73±20.24 | 100.04±21.10 | 0.820 |
| hs-CRP (mg/L) | 16.88 (5.00, 48.88) | 12.00 (3.50, 45.00) | 0.677 |

***WBC****: white blood cell;* ***RBC****: red blood cell;* ***NLR****: neutrophil-lymphocyte ratio;* ***LMR****: lymphocyte-monocyte ratio;* ***PLR****: platelet-lymphocyte ratio;* ***TBIL****: total bilirubin;* ***DBIL****: direct bilirubin;* ***AGR****: albumin–globulin ratio;* ***AST****: aspartate aminotransferase;* ***ALT****: alanine aminotransferase;* ***ALP****: alkaline phosphatase;* ***UA****: uric acid;* ***PTA****: prothrombin time activity;* ***hs-CRP****: hypersensitive C-reactive protein.*

**Figure S1.** Comparison of time-dependent ROC curves for random survival forests and XGBoost models. **A** 1-Year; **B** 3-Year; **C** 5-Year.


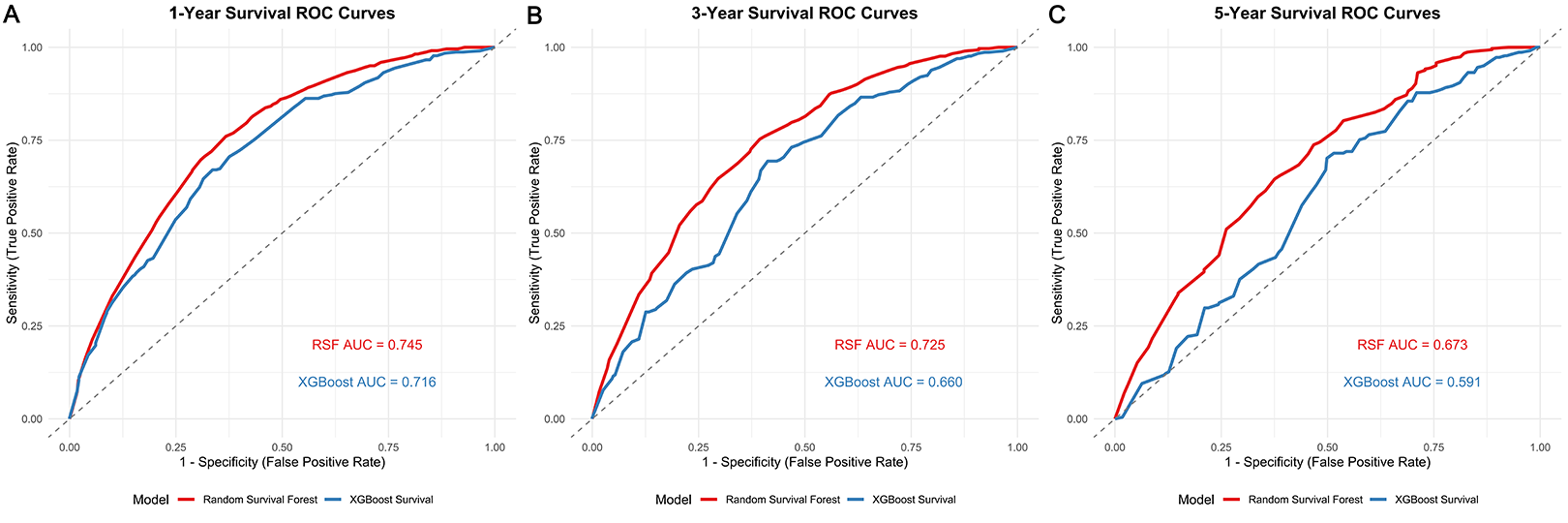


**Figure S2.** Feature importance based on Cox-LASSO model coefficients


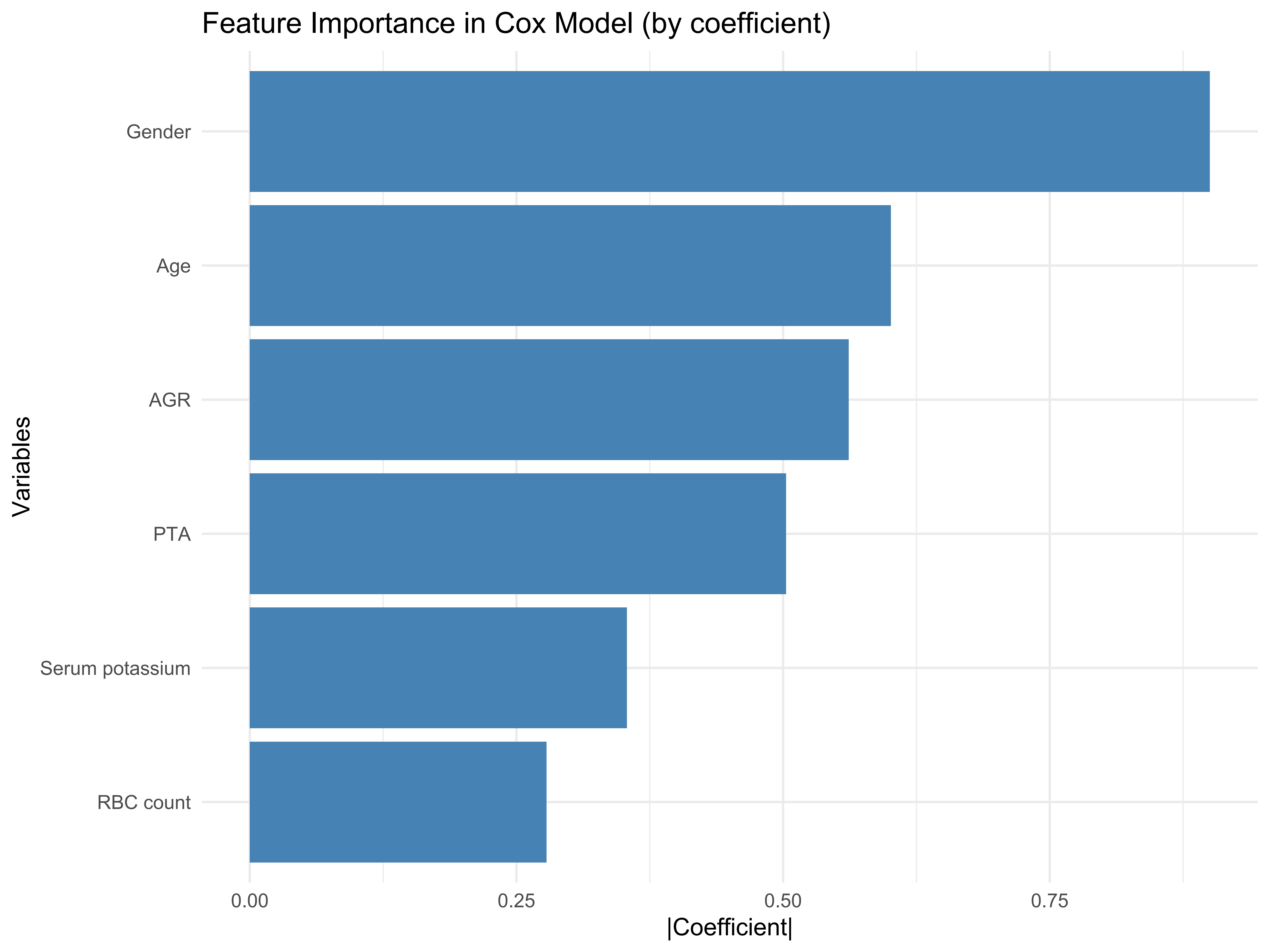


**Figure S3.** Sensitivity analysis of the predictive model across marital status subgroups. **A** Married/Living with partner; **B** Never married; **C** Widowed/Divorced/Separated.

**
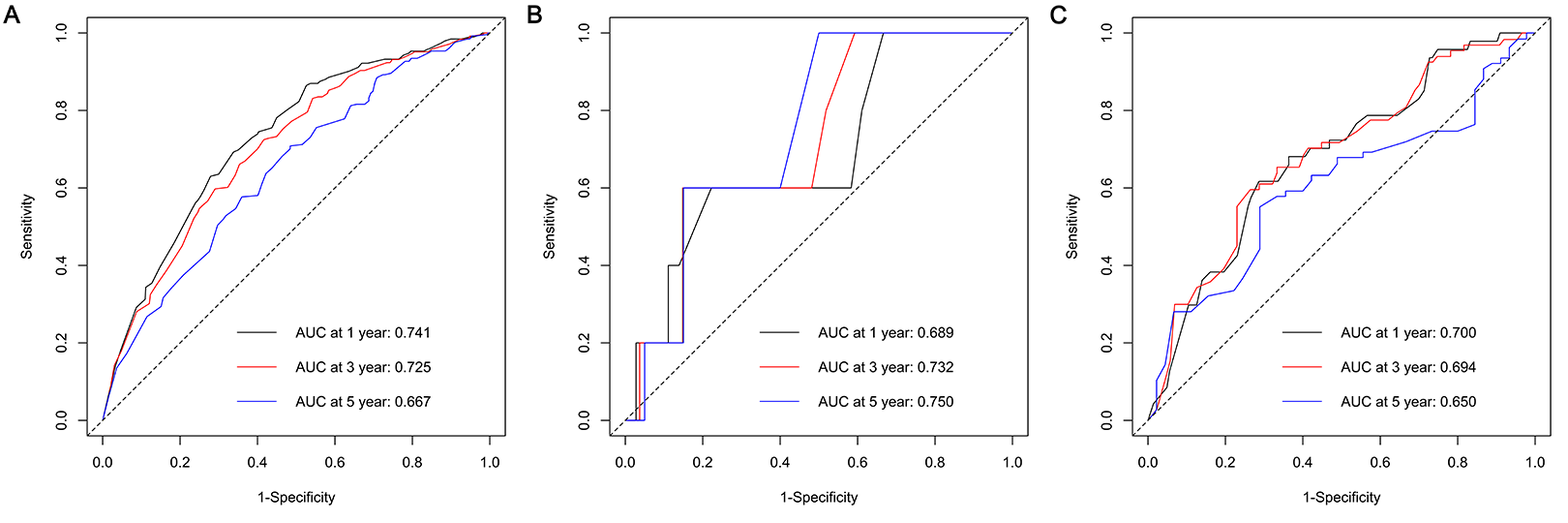
**

**Figure S4.** Sensitivity analysis of the predictive model after excluding patients with abnormal indicators


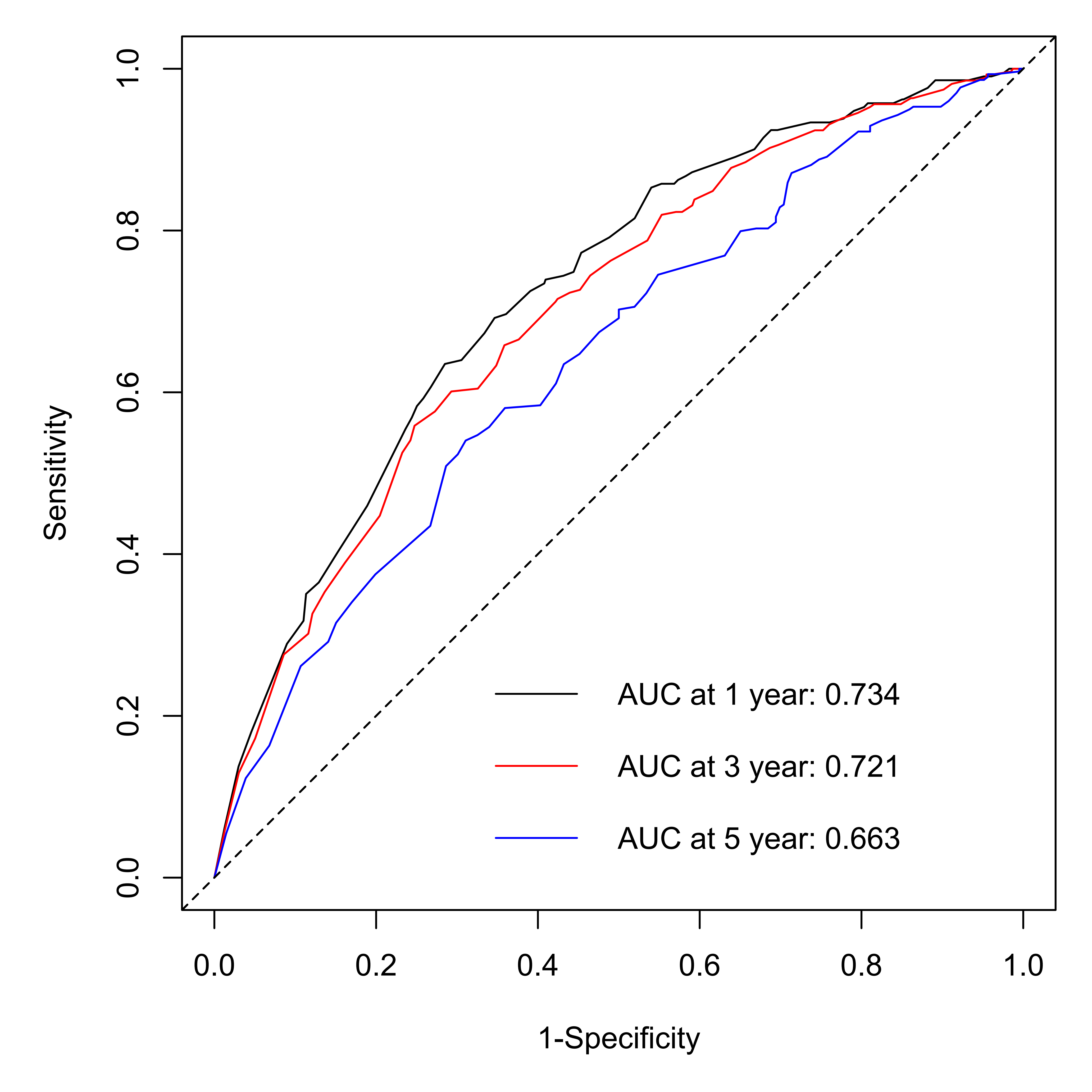

Supplement: Supplementary file 1 [file DataSheet1.docx]
